# Processing Demands Impact 3-Year-Olds’ Performance in a Spontaneous-Response Task: New Evidence for the Processing-Load Account of Early False-Belief Understanding

**DOI:** 10.1371/journal.pone.0142405

**Published:** 2015-11-12

**Authors:** Rose M. Scott, Erin Roby

**Affiliations:** University of California Merced, School of Social Sciences, Humanities, and Arts, Psychological Sciences, Merced, CA, United States of America; Birkbeck, University of London, UNITED KINGDOM

## Abstract

Prior to age four, children succeed in non-elicited-response false-belief tasks but fail elicited-response false-belief tasks. To explain this discrepancy, the processing-load account argues that the capacity to represent beliefs emerges in infancy, as indicated by early success on non-elicited-response tasks, but that children’s ability to demonstrate this capacity depends on the processing demands of the task and children’s processing skills. When processing demands exceed young children’s processing abilities, such as in standard elicited-response tasks, children fail despite their capacity to represent beliefs. Support for this account comes from recent evidence that reducing processing demands improves young children’s performance: when demands are sufficiently reduced, 2.5-year-olds succeed in elicited-response tasks. Here we sought complementary evidence for the processing-load account by examining whether increasing processing demands impeded children’s performance in a non-elicited-response task. 3-year-olds were tested in a preferential-looking task in which they heard a change-of-location false-belief story accompanied by a picture book; across children, we manipulated the amount of linguistic ambiguity in the story. The final page of the book showed two images: one that was consistent with the main character’s false belief and one that was consistent with reality. When the story was relatively unambiguous, children looked reliably longer at the false-belief-consistent image, successfully demonstrating their false-belief understanding. When the story was ambiguous, however, this undermined children’s performance: looking times to the belief-consistent image were correlated with verbal ability, and only children with verbal skills in the upper quartile of the sample demonstrated a significant preference for the belief-consistent image. These results support the processing-load account by demonstrating that regardless of whether a task involves an elicited response, children’s performance depends on the processing demands of the task and their processing skills. These findings also have implications for alternative, deflationary accounts of early false-belief understanding.

## Introduction

Adults routinely interpret the behavior of other individuals in terms of their underlying mental states. Developmental psychologists have long been interested in the origins of this fundamental ability. In particular, considerable research has focused on when children first understand that individuals can be mistaken, or hold false beliefs, about the world. Traditional investigations into this question used elicited-response false-belief tasks, in which children were asked direct questions about the likely behavior of an agent who held a false belief [1–3]. In one such task [1], children hear a story about Sally, who places a marble in a basket; in her absence, Anne moves the marble to a nearby box. Sally then returns and children are asked where she will look for her marble. Beginning at around age 4, children correctly answer that Sally will look in the basket, where she falsely believes the marble is located. In contrast, younger children indicate Sally will look in the box, suggesting a failure to represent her false belief. This widely replicated pattern of findings led to the conclusion that the capacity to attribute false beliefs to others did not emerge until at least age four [4–5].

However, recent evidence from non-elicited-response paradigms has challenged this conclusion. In these paradigms, children are not asked direct questions about the behavior of a mistaken agent. Instead, researchers assess children’s understanding of the agent’s false belief through a variety of alternative means. For instance, *spontaneous-response* tasks measure behaviors that children spontaneously produce as they observe a mistaken agent act in a scene, including where or how long they look at a scene [6–15], spontaneously pointing to inform the mistaken agent that something occurred in her absence [16–17], and tense facial expressions that suggest anticipation of the agent’s emotional reaction when she discovers her false belief [18]. In *prompted-action* tasks, children are prompted to perform an action such as choosing or a retrieving an object for an agent; in order to succeed, the children’s actions must be guided by an understanding of the agent’s false belief [19–23]. Finally, some paradigms assess *neurological* activity that occurs while observing an agent who holds a false belief [24–25]. Using these various non-elicited-response false-belief tasks, positive results have been obtained with children between 6 months and 3 years of age (for a review, see [26]).

These findings have raised a critical question: if young infants can succeed in non-elicited-response tasks, then why do children fail elicited-response false-belief tasks until at least age four? Together with our colleagues, we have proposed a *processing-load* account to explain this discrepancy between children’s performance on non-elicited- and elicited-response tasks [27–29]. This account, which builds on several prior accounts [30–34], makes two assumptions. First, like many researchers, we assume that the capacity to represent false beliefs emerges in infancy as indicated by children’s success on non-elicited-response tasks [6, 13–14, 19, 26, 35–36] (we return to alternative, deflationary accounts of these findings in the General Discussion). Second, whether children are able to demonstrate this capacity in a given false-belief task depends on both the processing demands of the task and children’s processing skills. If the processing demands exceed children’s processing abilities, then they will fail the task, despite their ability to represent beliefs.

According to this account, young children fail elicited-response false-belief tasks not because of an inability to represent beliefs but rather because these tasks generally impose greater processing demands than do non-elicited-response tasks. Specifically, when children are asked the test question in standard elicited-response tasks (e.g., “Where will Sally look for her marble?”), this activates a *response-selection* process: children must interpret the test question, choose to answer it, and select an appropriate response [29; 37–38]. Executing this response-selection process triggers a prepotent tendency to answer the question based on children’s own knowledge of reality and this response must be inhibited in order to answer correctly based on the agent’s false belief (*response-inhibition*) [30, 32, 39–42]. Finally, simultaneously holding in mind the agent’s false belief and executing the response-selection process imposes substantial demands on *working memory* [43–44]. These combined demands overwhelm the linguistic, inhibitory, and working memory abilities of young children, resulting in their failure in elicited-response tasks. The non-elicited-response tasks used to date have been designed to reduce or eliminate these various processing demands, allowing children to succeed at younger ages [27–29].

Support for the processing-load account comes from evidence that when processing demands are sufficiently reduced, children can pass elicited-response tasks well before age four [44–45]. For instance, Setoh et al. [44] devised an elicited-response task with reduced response-inhibition and response-selection demands. 2.5-year-old children heard a change-of-location false-belief story accompanied by a picture book: Emma placed an apple in one of two containers and in her absence, her brother Ethan found the apple and took it away. Removing the apple to an unknown location (as opposed to moving it to the other container) weakens the prepotent response evoked by the test question, thereby reducing response-inhibition demands [46]. Next, Emma returned to look for her apple. In the test trial, children saw two pictures, one of each container, and were asked where Emma would look for her apple. Interspersed with the story were two practice trials designed to reduce response-selection demands: children saw two pictures and were asked a “where” question (e.g., “Where is Emma’s apple?”) that required them to point to the matching picture. These trials thus gave children practice interpreting a “where” question and producing an appropriate response. With both the response-inhibition and response-selection demands reduced, children performed reliably above chance in the test trial, pointing to the container that Emma falsely believed held her apple. Additional experiments replicated these results and revealed that children failed if they received only one practice trial [44] or if the practice questions (“Which one is Emma’s apple?”) differed in linguistic form from the test question (“Where will Emma look for her apple?”) [47]. These results support the processing-load account by demonstrating that elicited-response tasks impose significant processing demands that easily overwhelm young children, but when these demands are sufficiently reduced children can succeed in elicited-response tasks at much younger ages, thereby revealing their underlying capacity to represent beliefs.

In the present research, we sought complementary evidence for the processing-load account by examining the impact of processing demands on children’s performance in a non-elicited-response task. If children’s ability to demonstrate their false-belief understanding depends on their ability to cope with the processing demands imposed by the task, then this should be true regardless of whether the task involves an elicited response. The processing-load account thus predicts that just as decreasing processing demands improves young children’s performance in elicited-response tasks, *increasing* processing demands should *impede* young children’s performance in non-elicited-response tasks. This account also predicts that when the demands of a non-elicited-response task are increased, children’s ability to demonstrate their false-belief understanding should be correlated with relevant processing skills, just as children’s performance in traditional elicited-response tasks is correlated with linguistic [48] and inhibitory control abilities [41]. Because research using non-elicited-response tasks has largely focused on minimizing processing demands in an effort to reveal early false-belief competence, to date no study has directly tested the impact of increasing processing demands on young children’s performance in such tasks.

However, a recent study by Schneider and colleagues [49] suggests that processing demands can impact adults’ performance in non-elicited-response tasks. Adult participants were tested in an anticipatory-looking task in which they saw three types of videos presented in random order: filler trials, false-belief trials, and true-belief trials. In filler trials, an agent watched as a puppet placed a ball on or in one of two boxes; a bell then rang and the agent reached for the ball. In false-belief trials, the puppet hid the ball in one box, moved it to the other box, and then the agent left. In the agent’s absence, the puppet returned the ball to its original box. The agent then returned, the bell rang, and the video paused for six seconds; during this window, participants’ looking time to each box was measured. True-belief trials were identical to false-belief trials except the agent left after the first hiding event and thus had a true belief the ball was in its original box. While watching the videos, all participants performed a secondary task. In the no-load condition, participants were told to press a button when they saw the agent wave at the puppet, which she did on some filler trials. In the low-load and high-load condition, participants were told to attend to a stream of letters spoken over headphones. Participants in the high-load condition were also told to count the number of 2-back repetitions of a letter (e.g., …A, B, A…) that occurred in each trial. Results indicated that participants in the no-load condition looked significantly longer at the empty box on false-belief trials than they did on true-belief trials, suggesting that they represented the agent’s false belief that the ball was in the empty box. This pattern did not occur in the low-load or high-load conditions, suggesting that the additional demands imposed by attending to the auditory stream interfered with participants’ ability to track the agent’s belief.

These findings demonstrate that engaging in a demanding secondary task impairs adults’ ability to anticipate the behavior of a mistaken agent. This suggests that, at least in adulthood, performance in a non-elicited-response task can be affected by processing demands. However, it remains unclear whether increasing the processing demands of a non-elicited-response task would impair young children’s performance. In addition, no study has directly tested whether children’s (or adults’) performance in high-demand non-elicited-response task is correlated with relevant processing skills, as predicted by the processing-load account.

Here we addressed these outstanding questions by manipulating the processing demands involved in a non-elicited-response false-belief task administered to 3-year-olds. Our experimental approach was motivated by two considerations. First, we sought to manipulate a processing demand that is known to impact young children’s performance in false-belief tasks. Many of the processing demands that have been systematically investigated in false-belief tasks are tied to answering direct questions about a mistaken agent and thus cannot be manipulated in a non-elicited-response paradigm. One promising exception is *linguistic ambiguity*, which has been shown to affect children’s performance in elicited-response tasks. Specifically, in order to correctly answer a test question such as “Where will Sally look for her marble?”, children must infer the experimenter’s intended meaning: where will Sally initially look for her marble, given her false belief about its location. Conversational and pragmatic factors [50] make this question subtly ambiguous: the experimenter could be asking where Sally ought to look for her marble, where she will eventually have to look in order to find the marble, or even for information about the marble’s current location [33–34, 51–52]. Young children sometimes interpret the question incorrectly, causing them to point to the marble’s actual location. Reducing the linguistic ambiguity of the task by making the intended meaning of the test question more apparent (e.g., “Where will Sally look *first* for her marble?”) improves young children’s performance [33–34, 51]. We reasoned that although the linguistic ambiguity in elicited-response tasks is embedded in the test question, it could easily occur at other points in a false-belief story, allowing us to manipulate linguistic ambiguity in a non-elicited-response task.

Second, we sought to use an established non-elicited-response task in which young children successfully demonstrate false-belief understanding. Because we intended to manipulate linguistic ambiguity, we required a task that involved a verbal false-belief story. We therefore chose to adapt the verbal preferential-looking task devised by Scott et al. [9], which took advantage of the well-established tendency for children and adults to look longer at images that match the sentences they hear [53]. In their task, 2.5-year-old children viewed a picture book while listening to a story about Emily. Emily placed her apple in one of two containers (a box or a basket) and then left. In her absence, her friend Sarah moved the apple to the other container. On the final page of the story, children saw two images: one in which Emily reached for the container that she falsely believed held her apple (original-container picture) and one in which she reached for the container that currently held the apple (current-container picture). While viewing these images, children heard, “Emily is looking for her apple.” Children looked reliably longer at the original- than the current-container picture, suggesting that they attributed to Emily a false belief that the apple was in its original container and, when they heard that Emily was looking for her apple, looked longer at the image in which she acted on this belief. These results demonstrate that in the absence of linguistic ambiguity, children succeed in a preferential-looking false-belief task.

In the present experiment, we examined how adding linguistic ambiguity to such a task impacted children’s performance. If the processing-load account is correct, then increasing linguistic ambiguity should interfere with children’s ability to demonstrate false-belief understanding in a preferential-looking task, and children’s ability to cope with this ambiguity should be correlated with their verbal skills.

## Method

### Participants

Fifty-six 3-year-olds (33.5–38.0 months, *M =* 35.5), 28 male and 28 female, participated in the study. An additional 2 children were tested but excluded, one because she failed to complete the experiment and one because the difference between his test looking times was over 3 standard deviations away from the mean. Equal numbers of males and females were randomly assigned to the ambiguous (*N* = 28, *M* = 35.3 months) and the control (*N* = 28, *M* = 35.6 months) condition of the false-belief task.

All participants were native English speakers. Parents were asked to identify their child’s race and ethnicity. In the ambiguous condition, 19 children were identified as White, 1 as Hawaiian or Pacific Islander, 1 as American Indian or Alaska native; 2 parents chose ‘other race’, 3 selected more than one race, and 2 chose not to respond. 7 of the children in the ambiguous condition identified as Hispanic or Latino, 18 identified as Not Hispanic or Latino, and 3 chose not to respond. In the control condition, 19 children were identified as White, 1 identified as Asian, 2 identified as African American, and 1 identified as American Indian or Alaska native; 3 parents chose ‘other race’, 1 selected more than one race, and 1 chose not respond. 11 of the children in the control condition identified as Hispanic or Latino, 14 identified Not Hispanic or Latino, and 3 chose not to respond.

We recorded the highest level of education reported by either parent. For children in the ambiguous condition, 3 parents completed high school, 9 completed an Associate’s degree, 10 completed a Bachelor’s degree, 4 completed a Master’s degree, and 2 completed a professional degree (MD or PhD). In the control condition, 9 parents completed high school, 2 completed an Associate’s degree, 9 completed a Bachelor’s degree, 6 completed a Master’s degree, and 2 completed a professional degree (MD or PhD).

The children’s names were obtained from birth records provided by the California Department of Public Health, as well as from a university maintained database of parents who had previously expressed interest in participating in research studies with their children. Parents were offered reimbursement for their transportation expenses and their child was given a small gift (book or t-shirt) for participating. Parents gave written informed consent for their child’s participation. The Institutional Review Board at the University of California Merced approved the protocol.

### Measures

#### Verbal ability

Children’s verbal ability was measured using the MacArthur-Bates Communicative Development Inventory, Level 3 (CDI-III) [54]. The CDI-III is a parental report measure with three subscales: Vocabulary, Sentences, and Using Language. The Vocabulary scale consists of a 100-item vocabulary checklist designed to assess children’s productive vocabulary; parents indicate any words that their child is able to produce. The Sentences scale assesses the complexity of children’s utterances using 12 sentence pairs. The sentences in each pair have similar meanings but differ in their grammatical level, with the second sentence being more sophisticated (e.g., “I like read stories” vs. “I like to read stories.”). For each pair, the parent is asked to indicate which sentence sounds *most* like the way their child currently talks; if their child’s utterances are even more sophisticated than those on the form, they are asked to select the second sentence. Children receive 1 point for each sentence pair for which they produce the second, more complex sentence. Sentence scale scores thus range from 0 to 12. The Using Language scale consists of 12 yes-or-no questions about the child’s language use, including comprehension (“Does your child understand the concept of ‘one’?”), semantics (“If you asked your child ‘What is a horse?’, could he answer ‘an animal’?”), and syntax (“Does your child give reasons for thing using the word ‘because’?”). Children receive one point for each *yes* answer, with scores ranging from 0 to 12.

#### False-belief task

Children were tested in a novel preferential-looking task in which they heard a change-of-location false-belief story accompanied by a picture book. The story contained seven story trials, two practice trials, and a single test trial (see S1 Appendix for complete pictures and script). On story trials, children saw a single image and heard a line of the story. On practice trials, children saw two images and were asked a question that required them to point to one of the two images. These trials familiarized children with the fact that some trials would involve two images and the story line would only match one of them; children were asked to point to encourage them to find the matching image whenever they saw a two-picture trial. In the test trial, children again saw two images and heard the final line of the story.

Children were randomly assigned to an ambiguous or a control condition. In the *ambiguous* condition, the story began by introducing Mia (story-1), who wanted to give her grandmother a cookie for her birthday (story-2). Children then received a practice trial (practice-1): they saw two images, one of Mia’s cookie and one of an orange, and were asked, “Where is Mia’s cookie?” Next, Mia put Grandma’s cookie into a blue bag (story-3; containers were counterbalanced across children) and went to put on her shoes (story-4). Children then received a second practice trial (practice-2): they saw two images, one of Mia’s shoe and one of a hat and were asked, “Where is Mia’s shoe?” While Mia was gone, her friend Danny took the cookie out of the bag (story-5). He placed the cookie in a pink box and left (story-6). Children then saw an image of Mia running into the room in a coat (story-7) and heard: “‘Hurry hurry,’ says Mia’s mom! ‘We’re leaving for Grandma’s!’ Mia puts on her coat and quickly runs in to get Grandma’s cookie.” This story line was ambiguous and open to two interpretations: (1) Mia runs in and hastily grabs the container that she believes contains the cookie (the bag; false-belief interpretation) and (2) Mia runs in, locates the cookie, and takes the container holding it (the box; reality interpretation). Children needed to determine which of these interpretations the experimenter intended. Although both interpretations were plausible, the false-belief interpretation was more appropriate for the story context (see S1 Adult Pilot).

Next, children received a test trial in which they saw two pictures of the back of an individual in a hooded coat. The identity of the individual was unknown. In one image, the individual carried the bag (original-container picture) and in the other, the individual carried the box (current-container picture). While viewing these images, children heard, “There’s Mia walking to Grandma’s. She’s carrying Grandma’s present.” We measured how long children looked at each of the pictures.

The *control* condition was identical to the ambiguous condition except that in story-7, children heard an additional sentence, “She grabs the cookie and runs out the door.” The purpose of this sentence was to make the intended meaning of the story apparent. By emphasizing the hurried nature of Mia’s actions, this sentence clarified that Mia selected a container without inspecting its contents, rendering the false-belief interpretation more likely. This additional sentence also gave children slightly more time (albeit only a few seconds) to process the experimenter’s intended meaning before the page was turned.

Note that the key difference between this false-belief task and the preferential-looking task used by Scott et al. [9] lay in the ambiguity of the language used to describe the main character’s actions when she returned to the room at the end of the story: in Scott et al.’s task the description was unambiguous, whereas in the ambiguous condition of our task the linguistic ambiguity of this description was increased. Increasing the linguistic ambiguity of the task in this fashion necessitated two additional differences between our task and Scott et al.’s paradigm. First, in the present task, children did not see the main character act on either container when she returned to the room–they instead had to infer which container she must have selected. Second, in Scott et al.’s task children saw two images on every story trial. For instance, in one story trial they heard “Emily is putting her apple in a box” while viewing an image of Emily placing her apple in a box and an image of Emily playing with blocks. All of the lines in the story trials of Scott et al. were unambiguous, and thus children readily identified the correct image on each trial and followed along with the story. Our task, however, included a story trial with an ambiguous description (story-7). Presenting two pictures on this trial would have created two levels of ambiguity–the interpretation of the experimenter’s sentence, and which image she was referring to. In order to avoid this additional layer of ambiguity, we chose to present only a single image on story trials and instead use practice trials to familiarize children with the two-picture format that they would encounter in the test trial. As will be clear from our results, neither of these additional differences appears to have interfered with children’s performance.

### Procedure

Children played freely with toys while their parents completed the consent form and CDI-III. They were then brought to an adjoining room, where they sat on their parent’s lap facing a table. On the table sat a wooden bookstand (56 × 53 cm; inclined at a 70° angle) that held a picture book. Pages of the book were attached to the top of the bookstand with multiple binder rings. Each page (56 × 28 cm) was composed of a large clear plastic photo sheet with plain, white paper backing; either one or two color photos (20 × 25 cm) were affixed to the each sheet. Single photos were centered, and double photos were placed 4.5 cm apart.

A camera centered behind the base of the bookstand captured the children’s eye-movements. A second camera mounted above and behind the child captured the stimuli and children’s physical actions. Parents were asked to remain quiet and neutral, and to close their eyes or look down to prevent them from biasing their children’s responses during the story.

The experimenter stood behind the book across from the child. To start, the book’s pages were face down behind the bookstand, away from the child. On each story trial, the experimenter turned a page towards the child, recited a line of the story, and then paused briefly, looking naturally between the book and the child. In each practice trial, the experimenter turned a page towards the child, asked the practice question, and then paused for up to 5 s. If children did not respond, the experimenter asked up to two additional times. The experimenter looked at the child during practice trials so that the child could not use her gaze as a cue for where to point. In the test trial, the experimenter turned the page, paused briefly, and then recited the final story line. During the test trial, the experimenter looked directly at the child to avoid giving gaze cues. Video footage was used to verify that all participants viewed the correct images and heard the correct story line on each trial.

The container in which Mia originally hid the cookie (and thus the container to which Danny moved it) and the sides of the current-container and original-container images in the test trial were counterbalanced across sex and condition.

### Coding

#### Verbal ability

Spearman correlations were calculated to examine relationships amongst the three subscales of the CDI-III. As shown in Table 1, the three subscales were significantly correlated with one another. We therefore summed the three scales to create a composite measure of children’s verbal ability. To explore the relationship between children’s verbal ability and their performance in the false-belief task, we divided children into quartiles (Q1, Q2, Q3, Q4) based on their verbal ability scores (see Table 2). In each condition, there were seven children in each quartile group. Preliminary analyses confirmed that neither verbal ability nor any of the subscales differed across the two conditions of the false-belief task, all *t*s < 1.

**Table 1:** Spearman correlations amongst subscales of the CDI-III.

Scale	Sentences	Using Language
Vocabulary	.80*	.78*
Sentences		.76*

N = 56

* *p* < .001 2-tailed

**Table 2:** Median (range) verbal ability scores, separately by condition and quartile.

	Ambiguous condition	Control condition	Overall
Q1	26 (13–43)	29 (16–47)	27.5 (13–47)
Q2	66 (55–73)	61 (48–75)	63.5 (48–75)
Q3	85 (78–98)	83 (76–97)	83.5 (76–98)
Q4	107 (103–121)	106 (103–117)	106.5 (103–121)
Overall	75.5 (13–121)	75.5 (16–117)	75.5 (13–121)

Possible scores ranged from 0 to 124.

#### False-belief task

For each practice trial, two independent coders indicated whether the child pointed to the matching image (practice-1: cookie, practice-2: shoe). Coders agreed on 98% of coded trials (Cohen’s kappa = .94, *p* < .001); disagreements were resolved by a third coder. Preliminary analyses indicated that the number of correct responses differed significantly from chance, χ^2^(2) = 64.54, *p* < .001: 47 children answered both questions correctly, 5 children answered one question correctly, and 4 answered neither correctly. Performance differed marginally across conditions in the first practice trial, *U* = 308, *p* = .051 (control: 27/28 correct; ambiguous: 21/28 correct), but not the second practice trial, *U* = 350, *p* = .352 (control: 27/28; ambiguous 24/28). The number of practice questions answered correctly did not differ across verbal ability groups in the overall sample, χ^2^(3) = 4.54, *p* = .226, or within condition, ambiguous χ^2^ (3) = 5.68, *p* = .136, control χ^2^ (3) = 3.00, *p* = .392.

In the test trial, we coded children’s looking behavior during the first six seconds that the pictures were visible; this test window was selected to match the six-second window used in Scott et al.’s [9] preferential-looking false-belief task. During this window, we coded where children looked frame-by-frame: left picture, right picture, experimenter, or away. All children were then coded from silent video by a second coder who did not know which was the original-location container. The two coders agreed on 95% of coded frames (Cohen’s kappa = .93, *p* < .001).

We computed children’s looking time in seconds to the original-container picture and the current-container picture; these looking times served as the dependent variables in our primary analyses. In addition, we calculated a preference score by subtracting children’s looking time to the current-location picture from their looking time to the original-location picture. The resulting preference score reflected the magnitude of children’s preference for the false-belief interpretation (positive scores) or the reality interpretation (negative scores) of the story.

## Predictions

Given children’s tendency to look longer at images that match sentences they hear (e.g., [9]), we expected that in the test trial children would look longer at the individual that they thought was Mia carrying Grandma’s present. However, children never saw which container Mia selected. In order to determine which individual was Mia, children had to infer which container she must have selected based on the story. Thus, children who arrived at the more appropriate false-belief interpretation of the story should look longer at the original-container than the current-location picture, whereas those who arrived at the less appropriate reality interpretation should instead look longer at the current-container picture.

We predicted that children’s ability to arrive at the more appropriate false-belief interpretation would depend on the linguistic ambiguity of the task. Based on the results of Scott et al. [9], we expected that when the intended meaning of the story was apparent, children would successfully demonstrate their understanding of Mia’s false belief. Thus, children in the control condition should show a clear preference for the false-belief interpretation, looking significantly longer at the original- than the current-container picture in the test trial. In contrast, we predicted that the presence of linguistic ambiguity would impede children’s performance, just as linguistic ambiguity interferes with children’s performance in elicited-response tasks: relative to children in the control condition, children in the ambiguous condition would be less likely to demonstrate a preference for the original-container picture. Finally, we predicted that when confronted with linguistic ambiguity, children’s performance would depend on their linguistic ability: children with more advanced verbal skills would be more likely to arrive at the false-belief interpretation. Thus, we expected that in the ambiguous condition, children’s preference for the false-belief interpretation would be correlated with their verbal ability.

Might performance in the control condition also be correlated with verbal ability, given the verbal nature of the task? Although possible, we did not predict such a correlation in the control condition. This was based on the performance of children in Scott et al.’s [9] original study: across conditions, 79% of children looked longer at the belief-consistent image. Although Scott et al. [9] did not directly assess verbal ability, it seems unlikely that children’s performance in their task was strongly dependent on verbal skills, given this high success rate. Thus, we predicted similar findings in the present experiment: when the intended meaning of the story was apparent, children’s performance would not depend on their verbal ability.

## Results

Preliminary analyses of children’s looking times to the original- and current-container pictures in the test trial revealed no interactions of picture and verbal ability with sex, number of correct responses in the practice trials, which container Mia originally placed the cookie in, or the side of the original-container picture in the test trial, all *F*s < 1.06, all *p*s > .36. These factors were not examined further.

**Fig 1:**
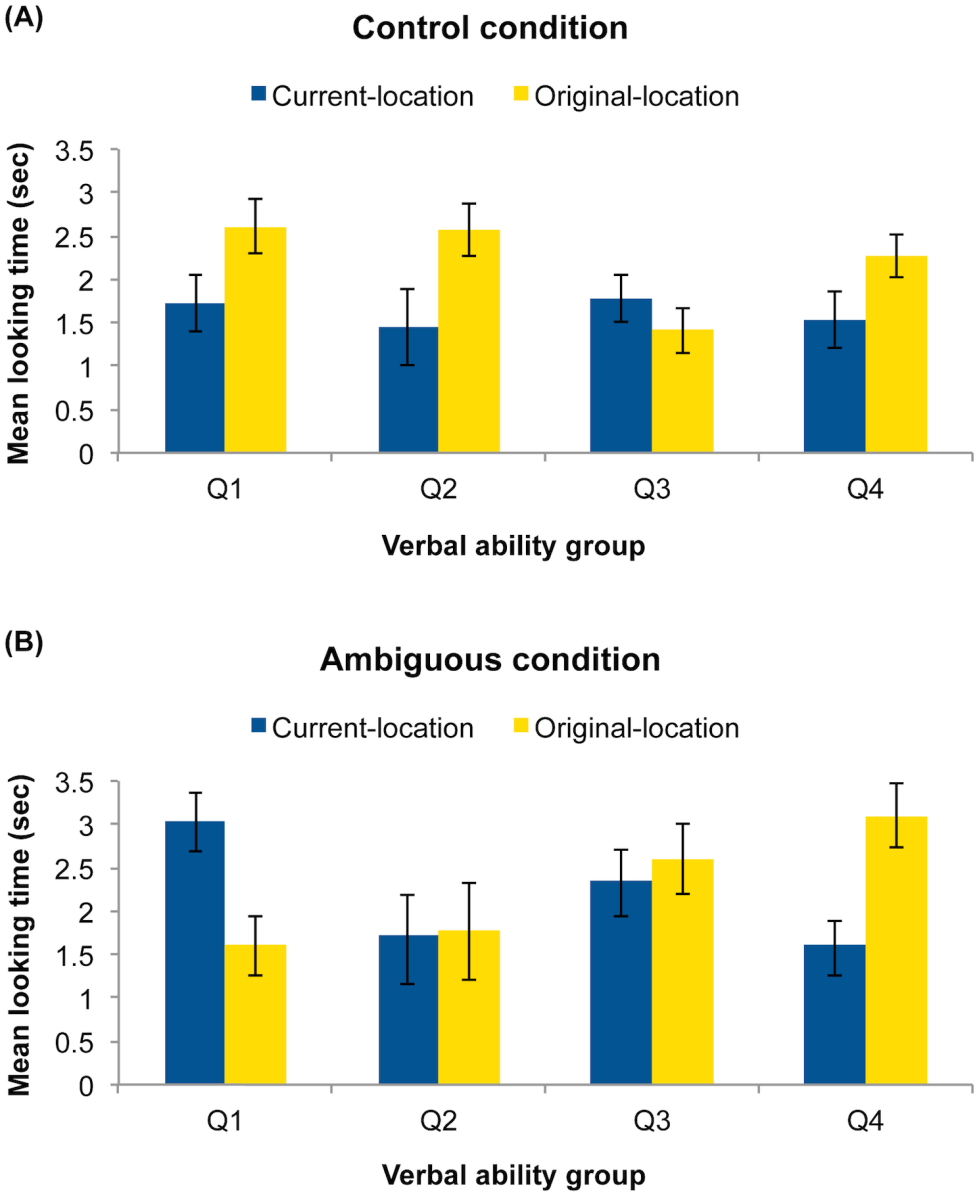
Mean looking time (s) to the original-location and current-location pictures in each verbal ability group. Looking times are shown separately for the control condition (A) and the ambiguous condition (B). Error bars represent standard errors.

Children’s looking times to the two pictures during the test trial (see Fig 1) were analyzed using an analysis of variance (ANOVA) with picture (original-container, current-container) as a within-subject factor and condition (ambiguous, control) and verbal ability group (Q1, Q2, Q3, Q4) as between-subjects factors. This analysis revealed a significant three-way interaction of picture, condition, and verbal ability, *F*(3, 48) = 3.20, *p* = .032, η^2^ = .17, indicating that, as predicted, looking times to the two images varied as a function of ambiguity and verbal ability. The analysis also revealed marginal effects of picture, *F*(1, 48) = 2.81, *p* = .10, η^2^ = .055, and condition, *F*(1, 48) = 3.99, *p* = .051, η^2^ = .08, as well as marginal interaction of picture with verbal ability, *F*(3, 48) = 2.38, *p* = .081, η^2^ = .13, and a marginal interaction of condition with verbal ability, *F*(3, 48) = 2.52, *p* = .069, η^2^ = .14. No other effects were significant, all *F*s < 1.52, all *p*s > .22. To further explore the significant three-way interaction, we next examined the two conditions separately.

### Control condition

**Fig 2:**
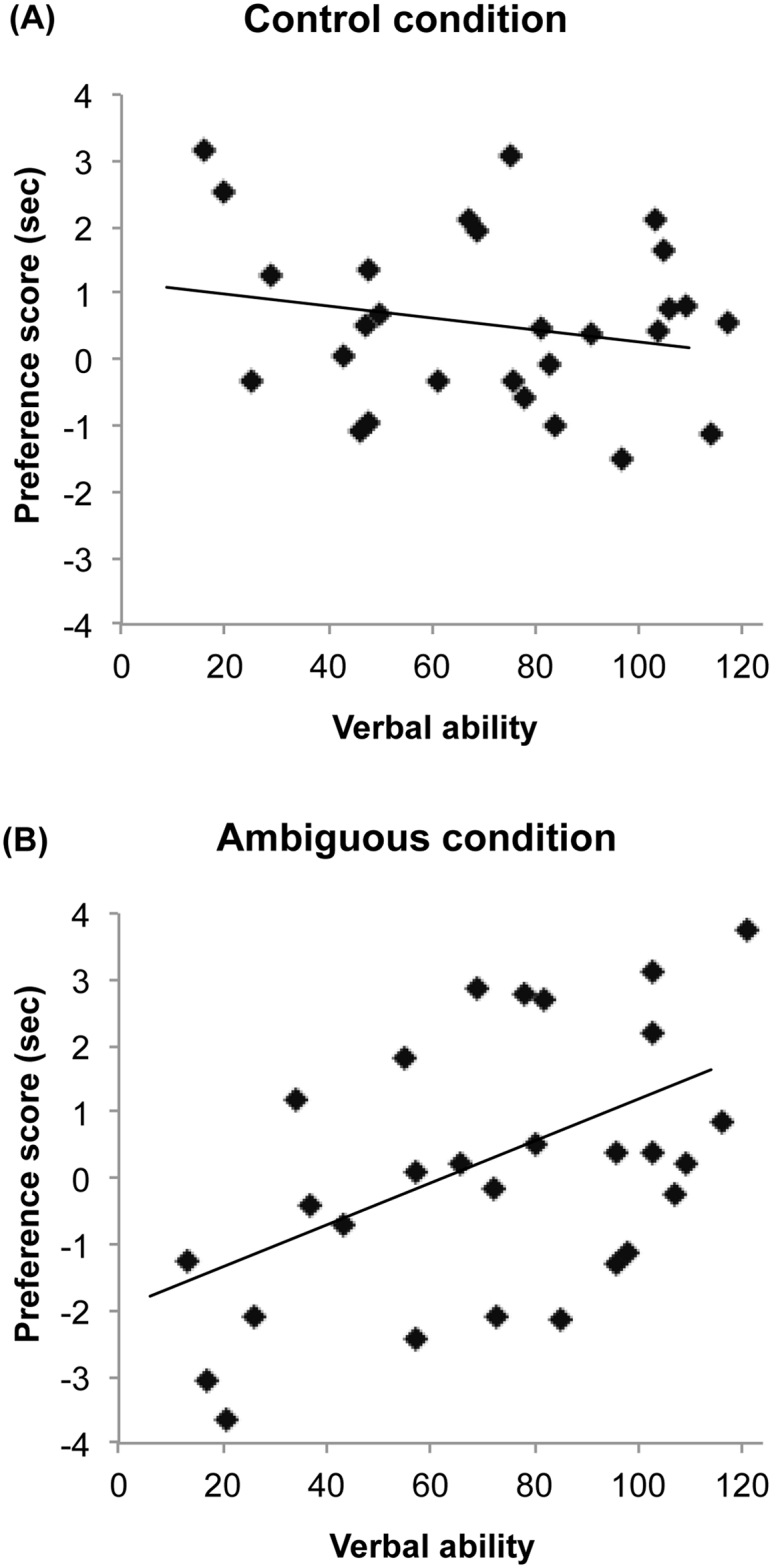
Relationship between preference scores and verbal ability in the control (A) and ambiguous conditions (B). For preference scores, positive values indicate a preference for the false-belief interpretation and negative values indicate a preference for the reality interpretation. Solid lines indicate the linear trend.

An ANOVA on children’s looking times during the test trial with picture (original-container, current-container) and verbal ability group (Q1, Q2, Q3, Q4) as a factor revealed a significant effect of picture, *F*(1, 24) = 6.57, *p* = .017, η^2^ = .22. Children looked significantly longer at the original-container picture (*M* = 2.21, *SD* = .86) than at the current-container picture (*M* = 1.62, *SD* = .88) (see Fig 1A). There was no significant effect of verbal ability, *F*(3, 24) = 1.23, *p* = .320, and no interaction of picture and verbal ability, *F*(3, 24) = 2.05, *p* = .134, η^2^ = .20, indicating that in this condition, children’s performance did not vary as a function of their verbal ability. Confirming this result, children’s preference scores were not significantly correlated with their verbal ability scores, *r*_*s*_(28) = -.16, *p* = .421 (see Fig 2A). Thus, as predicted, when the linguistic ambiguity of the task was low, children successfully demonstrated an understanding of Mia’s false belief by showing a clear preference for the false-belief interpretation of the story.

### Ambiguous condition

An ANOVA on children’s looking times during the test trial with picture (original-container, current-container) and verbal ability group (Q1, Q2, Q3, Q4) as a factor revealed no effect of picture, *F* < 1. As a group, children in the ambiguous condition looked equally at the original-container (*M* = 2.27, *SD* = 1.23) and current-container (*M* = 2.18, *SD* = 1.08) pictures, suggesting that the presence of linguistic ambiguity undermined their ability to demonstrate an understanding of Mia’s false belief. However, there was a significant interaction of picture and verbal ability, *F*(3, 24) = 3.14, *p* = .044, η^2^ = .28, indicating that, as predicted, children’s performance in this condition varied as a function of their verbal ability. There was also a marginal effect of verbal ability, *F*(3, 24) = 2.37, *p* = .096, η^2^ = .23, reflecting the fact that children in Q2 exhibited slightly shorter looking times (*M* = 1.74, *SD* = .96) than children in the other three groups (Q1: *M* = 2.32, *SD* = .34; Q3: *M* = 2.47, *SD* = .29; Q4: *M* = 2.36, *SD* = .35).

Planned comparisons revealed that children in the lowest quartile of verbal ability (Q1) looked significantly longer at the current-container (*M* = 3.03, *SD* = .88) than at the original-container (*M* = 1.60, *SD* = .91) picture, *F*(1, 24) = 4.55, *p* = .043, Cohen’s *d* = 1.60, demonstrating a preference for the reality interpretation. In contrast, children in the highest quartile (Q4) looked significantly longer at the original-container (*M* = 3.10, *SD* = .97) than at the current-container (*M* = 1.62, *SD* = .73) picture, *F*(1, 24) = 4.88, *p* = .037, *d* = 1.72, exhibiting a preference for the false-belief interpretation. Children in the middle two quartiles looked relatively equally at the two images, both *F*s < 1 (Q2: original-container *M* = 1.77, *SD* = 1.47, current-container *M* = 1.72, *SD* = 1.23; Q3: original-container *M* = 2.60, *SD* = 1.05, current-container *M* = 2.34, *SD* = .97). Confirming this pattern, children’s preference scores were strongly correlated with their verbal ability, *r*_*s*_(28) = .48, *p* = .010, with children with more advanced verbal skills exhibiting stronger preferences for the false-belief interpretation of the story (see Fig 2B).

These results suggest that the linguistic ambiguity in the ambiguous condition impeded children’s ability to demonstrate false-belief understanding. Children’s ability to overcome this ambiguity and arrive at the contextually appropriate false-belief interpretation depended on their verbal skills, with only the most verbally advanced children (Q4) demonstrating a significant preference for the false-belief interpretation.

### An effect of verbal ability or executive function?

An anonymous reviewer suggested a possible alternative explanation for the observed differences in test performance across conditions. Recall that on the first practice trial, children in the ambiguous condition were marginally less likely to respond correctly than those in the control condition. This difference could indicate that the children in the two conditions differed in their executive function abilities. If so, the poorer test performance of children in the ambiguous condition could reflect an inability to cope with the executive demands of the task. Note that if this were the case, our results would still support the processing-load account by demonstrating a relationship between children’s processing skills and their performance in a high-demand spontaneous-response task.

To address this alternative explanation, we examined whether children’s performance in the test trial was related to their performance in the practice trials. The number of correct responses in the practice trials was not significantly correlated with children’s preference for the false-belief interpretation in the overall sample, *r*_*s*_(56) = .198, *p* = .145, or in either condition, ambiguous *r*_*s*_(28) = .195, *p* = .320, control *r*_s_(28) = .226, *p* = .247. This suggests that children’s performance in the test trial did not vary as a function of their performance in the practice trials. An analysis of covariance (ANCOVA) with picture (original-container, current-container) as a within-subject factor, condition (ambiguous, control) and verbal ability group (Q1, Q2, Q3, Q4) as between-subjects factors, and number of practice trials correct as a covariate again revealed a significant three-way interaction of picture, condition, and verbal ability, *F*(3, 47) = 2.83, *p* = .048, η^2^ = .15. Thus, our predicted difference across conditions and verbal ability groups held, even after controlling for practice performance. Finally, partial correlations controlling for the number of practice trials answered correctly revealed the same patterns as above: children’s verbal ability was significantly correlated with their preference for the false-belief interpretation in the ambiguous condition, *r*_*s*_(25) = .46, *p* = .016, but not in the control condition, *r*_*s*_(25) = -.20, *p* = .323.

Given that we did not measure children’s executive function abilities directly, we cannot rule out the possibility that children in the two conditions differed in their executive function skills. However, these results provide no evidence that children’s performance in the test trial was related to their performance in the practice trials, nor that our effects were carried by children who were unable to answer the practice trials correctly. Thus, we find it more plausible that the effects we observed were related to children’s verbal ability and the linguistic ambiguity we manipulated.

## General Discussion

The processing-load account argues that while the capacity to represent false beliefs emerges early in infancy, whether children successfully demonstrate this capacity in a false-belief task depends on the processing demands of the tasks and children’s ability to cope with those demands. In the present experiment, we tested this account by examining the impact of processing demands on children’s performance in a non-elicited-response task. 3-year-olds were tested in a preferential-looking task in which they heard a change-of-location false-belief story. Across children, we manipulated the linguistic ambiguity of the story. When the story was relatively unambiguous, children demonstrated an understanding of the main character’s false belief. When the story contained linguistic ambiguity, however, children’s performance depended on their verbal ability, and only those children with the most advanced verbal abilities successfully demonstrated false-belief understanding.

These findings expand our understanding of early false-belief reasoning in several ways. First, the results of the ambiguous condition provide the first experimental demonstration that high processing demands impede young children’s performance in non-elicited-response tasks. Together with the results of Schneider et al. [49], these findings suggest that performance in non-elicited-response tasks can be affected by processing demands across the lifespan. Moreover, the correlation between children’s performance in the ambiguous condition and their verbal ability demonstrates that when confronted with a high-demand non-elicited-response task, children’s ability to pass the task depends on their processing skills. These results thus confirm both of the predictions of the processing-load account regarding the impact of processing demands on children’s performance in non-elicited-response tasks. These findings also complement recent evidence that decreasing processing demands improves young children’s performance in elicited-response tasks [44–45]. Together, these two sets of findings provide strong support for the processing-load account by establishing that regardless of a whether or not they need to produce an elicited response, children’s ability to demonstrate their false-belief understanding depends on the processing demands of the task and their processing skills.

Second, the positive results in the control condition replicate and extend the findings reported by Scott et al. [9]. Recall that in the Scott et al.’s task, children heard that the main character, Emily, was looking for her apple while viewing images that depicted Emily searching in the apple’s original or current location. In order to succeed, children needed to recognize which image corresponded to Emily’s likely search behavior, given her false belief. In the present task, children did not see which container the main character selected. Instead, children simply saw an image of Mia approaching the two containers and then received a test trial in which they saw two anonymous individuals, each carrying one of the two containers. In order to succeed in this task, children had to infer which container Mia must have selected and then use this information to identify which of the individuals in the test trial was likely to be Mia. Children in the control condition succeeded, despite this additional layer of inference (this also suggests that reducing the number of two-picture trials in the task did not impede children’s performance). These positive results thus add to a growing body of evidence (reviewed in the Introduction) that the capacity to represent false beliefs is present before age four.

Third, the contrast between the results of the ambiguous and the control condition underscores that very subtle changes to a false-belief task can impact children’s ability to demonstrate their false-belief understanding. These two conditions differed by only a single sentence, “She grabs the cookie and runs out the door.” With this sentence, children succeeded; without it, performance was strongly correlated with verbal ability and only the most advanced children were able to succeed. Similar effects of subtle manipulations have been observed in elicited-response tasks. For instance, simply adding *first* to the standard test question improves 3.5-year-olds’ performance in elicited-response tasks [33–34]. As described in the Introduction, 2.5-year-olds can pass a low-inhibition elicited-response task that involves two practice trials to reduce response-selection demands, but they fail if they receive only one practice trial or if the practice questions differ in linguistic form (e.g., “Which one is Emma’s apple?”) from the test question [44, 47]. The fact that slight variations can dramatically affect children’s performance in both non-elicited-response and elicited-response tasks suggests that negative results in false-belief tasks should be interpreted with caution, as such failures could stem from aspects of the task that interfere with performance rather than indicating an underlying conceptual deficit [55].

### Alternative accounts of early false-belief understanding

We have argued that the positive results obtained with infants and toddlers in non-elicited-response tasks indicate that the capacity to represent false beliefs emerges in infancy. However, a number of researchers maintain that the capacity to represent false beliefs does not emerge until age four, as indicated by success on elicited-response tasks, and offer alternative, deflationary accounts for the results of non-elicited-response false-belief tasks [56–62]. Although the present experiment was designed to test predictions from the processing-load account, our results also bear on several of these alternative accounts, which we discuss next.

#### Low-level process and behavioral-rule accounts

The low-level process account [58–59] argues that the results of non-elicited-response tasks do not provide evidence of a capacity to represent mental states. Instead, these tasks are assumed to draw on domain-general processes of perception, attention, and memory. In order to explain the results of spontaneous-response tasks that assess looking time, such as the preferential-looking task used in the present experiment, the low-level process account makes two assumptions. First, children look longer at events that, relative to other recent events, have novel spatiotemporal relations among actions and objects. Second, children sometimes suffer from retroactive interference: salient events (such as the return of an agent) distract children, causing them to forget other recent events. Children therefore look longer at events that are novel or that seem novel due to retroactive interference. Heyes [58] has argued that in all looking-time tasks used to date, responses to perceptual novelty and responses based on the agent’s mental states were conflated, giving rise to apparent false-belief understanding (for critical discussion of this claim, see [63]). For instance, this account argues that when an agent places an object in location-A and it is moved to location-B in her absence, children look longer if the agent reaches to location-B because this is perceptually novel (she has never reached to B before) rather than because this is inconsistent with her false belief [8].

The behavioral-rule account [60, 62] offers a different non-mentalistic explanation for children’s success in non-elicited-response tasks. According to this account, non-elicited-response tasks assess children’s expectations about behavior rather than their understanding of mental states. In everyday life, children gather information, in the form of statistical regularities or behavioral rules, about how agents typically behave in particular situations. When children observe an agent in one of these situations in a laboratory task, they retrieve the appropriate behavioral rule and use it to interpret or predict the agent’s actions. For instance, it has been argued that infants expect agents to look for objects where they last saw them [64] and that this expectation gives rise to a variety of responses in non-elicited-response tasks [62] such as anticipatory looks towards the location where an agent last saw an object [13], and looking longer when an agent searches for an object in a location other than where she last saw it [8, 14].

Could either of these accounts explain the present findings? In order to do so, they would need to address why children in the two conditions responded differently. If one assumes that the current-container picture was the more perceptually novel image in the test trial (because Mia had never acted on that container before), then children in both conditions should have looked longer at the current-container picture. If one instead assumes that the original-container picture was novel (though it is not clear why that would be the case, even factoring in potential retroactive interference caused by Mia’s return), then children in both conditions should have looked longer at the original-container picture. In either case, the two conditions should have produced comparable results. Similarly, if we assume that infants expect agents to look for objects where they last saw them, then presumably all of the 3-year-olds in the present experiment possessed this expectation as well. Observing Mia’s actions should have activated this behavioral rule for children in both conditions, leading to similar patterns of responding in the test trial. Thus, the challenge for these accounts is to explain why altering a single sentence in the story affected what children found novel in the test trial (low-level process account) or the activation of a behavioral rule (behavioral-rule account). More critically, one would have to explain why responses in the ambiguous, but not the control condition, were correlated with verbal ability. While we cannot rule out the possibility that such an explanation exists, it is not immediately obvious why verbal ability would alter what children found perceptually novel, nor why 3-year-olds would require advanced verbal abilities to draw on a behavioral rule that is readily activated by preverbal infants when viewing an agent’s actions.

The low-level process account and the behavioral-rule account have both been offered as post hoc explanations for success in non-elicited-response tasks (see [36, 65]). However, the observed differences between conditions in the present experiment call for an account that can offer a coherent, integrated explanation for both success and failure in non-elicited-response tasks. Neither the low-level process account nor the behavioral-rule account offers such an explanation at present. Our results thus cast doubt on these accounts in their current forms.

#### Minimalist account

According to the minimalist account [56–57], humans possess two systems for reasoning about the behavior of agents. The late-developing system, which emerges at around age four, is capable of representing false beliefs. This system is highly flexible, allowing children and adults to represent any belief that they themselves can entertain. But this flexibility comes at the cost of efficiency: the late-developing system is slow, effortful, and dependent on language and executive function resources. The early-developing system, which is present in infancy, cannot represent beliefs as such and instead tracks simpler belief-like registrations: an agent who encounters an object registers its location and properties. The early-developing system can use registrations to predict and interpret an agent’s future actions in some situations (e.g., an agent will search for an object where she last registered it). However, because the early-developing system tracks registrations instead of genuine beliefs, its performance is subject to a number of ‘signature limits,’ such as an inability to handle situations involving false beliefs about identity [55]. This limited flexibility is offset by efficiency: because the system represents simpler states, it is fast, automatic, and operates independent of language and executive function.

Advocates of the minimalist account have suggested that the late-developing system is required for successful performance on elicited-response tasks because these tasks require explicit judgments about beliefs [56]. In contrast, the early-developing system is responsible for “guiding children’s eye movements” in non-elicited-response tasks that assess eye gaze, such as the one used in the present research (p. 964 in [56]). This predicts that performance on these tasks should be independent of language and executive function. The present findings are inconsistent with this prediction: they demonstrate a clear link between verbal ability and children’s performance on a non-elicited-response task that measured gaze. The fact that adults’ anticipatory-looking responses depend on executive demands [49] also seems inconsistent with the predictions of the minimalist account.

There are several ways these results could potentially be reconciled with the minimalist account. One possibility is that tasks that measure eye gaze do not always assess the early-developing system. Perhaps the present task instead required the late-developing system and this is why performance was correlated with verbal ability. If that were the case, however, it would suggest that the late-developing system emerges by three years of age. A second possibility is that the present task did assess the early-developing system, but that the operation of this system is not as independent of language as previously thought. It may be that although the early-developing system does not obligatorily depend on language and executive function, it is also not entirely encapsulated from these abilities. A third issue concerns the time-course of the response that is measured in a particular non-elicited-response task. In both the present task and Schneider et al.’s [49] task, eye gaze was measured in a 6-second time window. In contrast, the recent studies that have been argued to show evidence of a limited, early-developing system have measured anticipatory looking in much shorter, 1.75-second windows [55, 66]. This raises the possibility that the timing of the response window may affect whether a task assesses the early- or late-developing system, demonstrates signature limits, or is affected by language or executive function.

As the preceding discussion makes clear, our results cannot rule out the possibility that humans possess two systems for psychological reasoning. However, our findings raise a number of important questions regarding the operation and measurement of the two systems proposed by the minimalist account that should be explored in future research.

### Conclusion

Our results provide new evidence in support of the processing-load account by demonstrating that children’s performance in false-belief tasks depends on processing demands and processing skills, regardless of whether the task involves an elicited response. Our findings are thus consistent with the claim that both elicited- and non-elicited-response tasks measure a capacity to represent beliefs, but that subtle manipulations, such as modifying a word or sentence, can improve or undermine children’s ability to demonstrate this capacity.

## Supporting Information

S1 Adult Pilot Pilot experiment conducted with adult participants. (DOCX)

S1 Appendix Complete pictures and script for the ambiguous and control conditions of the false-belief task. (DOCX)
