# Supplementary material for: Processing Demands Impact 3-Year-Olds’ Performance in a Spontaneous-Response Task: New Evidence for the Processing-Load Account of Early False-Belief Understanding
Source: PLoS One. 2015 Nov 12;10(11):e0142405. doi: 10.1371/journal.pone.0142405 (PMC4642936; doi:10.1371/journal.pone.0142405)
Supplement: S1 Adult Pilot — (DOCX) [file pone.0142405.s001.docx]

**S1 Adult Pilot**

The story in the ambiguous condition was open to two interpretations: a false-belief interpretation and a reality interpretation. In order to determine which interpretation was more contextually appropriate, we piloted this condition with a sample of adult participants. Like children, adults demonstrate a well-established tendency to look at images that match spoken utterances and looking-time measures are widely used to assess language comprehension [e.g., 53]. Thus, measuring which image adults looked longer at in the test trial would indicate which individual they thought was Mia and thereby which interpretation of the story they viewed as more plausible.

**Method**

**Participants.** 16 adult participants (*M* = 20 years; range 18-25; 12 female) completed the experiment for course credit. All participants gave written informed consent, and the protocol was approved by the Institutional Review Board of the University of California Merced.

**Apparatus and Procedure.** Adults were tested in the false-belief task using the same apparatus and procedure as children in the ambiguous condition. Prior to starting the experiment, participants were told that they would hear a story used in experiments with children. They were asked to respond naturally throughout the story and to pay close attention because they could be asked questions about the story after it was finished.

**Coding.** We coded where adults’ looked (left picture, right picture, experimenter, away) frame-by-frame during the first six seconds that the pictures were visible. All participants were then coded from silent video by a second coder who did not know which was the original-location container. The two coders agreed on 94% of coded frames. We then computed adults’ looking time in seconds to the original-container picture and to the current-container picture.

**Results**

An analysis of variance (ANOVA) with picture (original-container, current-container) as a within-subject factor revealed a main effect of picture, *F*(1, 15) = 4.56, *p* = .05, η^2^ = .23. Participants looked reliably longer at the original-container picture (*M* = 2.96, *SD* = 1.07) than the current-container picture (*M* = 2.06, *SD* = .89). This suggests that adult listeners interpret the story as Mia running in and grabbing the container that she falsely believed held the cookie. Thus, the false-belief interpretation of the story is the more plausible (and more adult-like) interpretation.
