# Supplementary material for: Processing Demands Impact 3-Year-Olds’ Performance in a Spontaneous-Response Task: New Evidence for the Processing-Load Account of Early False-Belief Understanding
Source: PLoS One. 2015 Nov 12;10(11):e0142405. doi: 10.1371/journal.pone.0142405 (PMC4642936; doi:10.1371/journal.pone.0142405)

**S1 Appendix: Pictures and Script used in the False-belief Task**

Ambiguous Condition


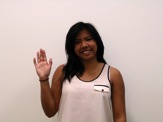


**Story-1**

“This is a story about a girl named Mia. Look! There’s Mia!”

**Story-2**


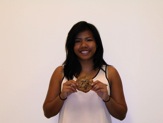


“Mia has a cookie. It’s her Grandma’s birthday and Mia wants to give her a cookie.”

**Practice-1**


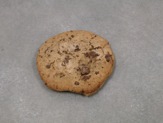

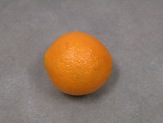


“Where is Mia’s cookie?”

**Story-3**

“Mia puts the cookie in a blue bag.”


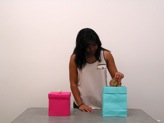

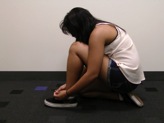


**Story-4**

“Then Mia goes to put on her shoes.”

**Practice-2**


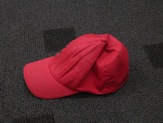

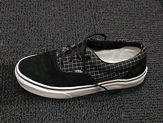


“Where is Mia’s shoe?”

**Story-5**

“While Mia is gone, her friend Danny takes the cookie out of the blue bag.”


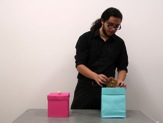

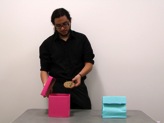


**Story-6**

“Danny puts the cookie in a pink box. And then he leaves.”

Control Condition

**Test trial**


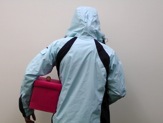

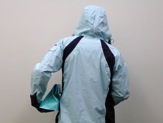


“There is Mia walking to Grandma’s! She’s carrying Grandma’s present.”

**Story-7**

“Hurry, Hurry! Says Mia’s Mom. We’re leaving for Grandma’s! Mia puts on her coat, then she quickly runs in to get Grandma’s cookie.”


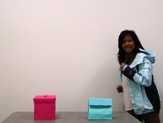


The control condition was identical to the ambiguous condition except for the script used in Story-7. In the control condition, this trial included an additional sentence, “She grabs the cookie and runs out the door.”

**Story-7**

“Hurry, Hurry! Says Mia’s Mom. We’re leaving for Grandma’s! Mia puts on her coat, then she quickly runs in to get Grandma’s cookie. She grabs the cookie and runs out the door.”


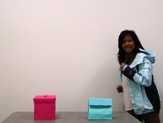

Supplement: S1 Appendix — (DOCX) [file pone.0142405.s002.docx]
